# Supplementary material for: Integrating interferon-gamma release assay testing into provision of tuberculosis preventive therapy is feasible in a tuberculosis high burden resource-limited setting: A mixed methods study
Source: PLOS Glob Public Health. 2022 Jul 6;2(7):e0000197. doi: 10.1371/journal.pgph.0000197 (PMC10022101; doi:10.1371/journal.pgph.0000197)
Supplement: S1 Text — (DOCX) [file pgph.0000197.s002.docx]

**Focus group discussion/ Key informant guide for healthcare worker study participants**

**Purpose:** provide questions to facilitate the collection of qualitative information on home-based IGRA test for LTBI diagnosis.

**Introduction provided by the facilitator**

- Organization(s) affiliated with activity
- Additional staff assisting or observing the discussion
- Activity short term purpose: To gather participants’ perspectives on lessons learnt and challenges met in the implementation of the home-based IGRA test for latent TB infection “hidden TB”.
- Activity long term purpose: To gather information to provide to the MOH on the adoption of new WHO recommended tests for “hidden TB” Infection.

**Ethical considerations**

- - Voluntary and anonymous participation with no names recorded and individual level information not shared.
  - You can choose to stop participating at any time. We expect not to spend more than 90 minutes during this discussion.
  - We will take notes during the discussion and will audio record when possible, to help capture your comments accurately and complete our notes. We will destroy the recordings after we make the notes.
  - Ask permission to take photos or record session.

**Introduction provided by the participants**

- Each participant should describe their background as it relates to the design of the focus group discussion.
- For example:
  - If the facilitator is talking with some group/ individual home visitors, clinicians, lab technicians etc.

**Discussion guide**

1. How important or unimportant is the home-based (IGRA) test using a blood sample for “hidden tuberculosis” that has been done in this study?

(Probing questions)

- Importance to individuals?
- Importance to family
- Importance to community
- Importance to health system?
- Importance to control of Tuberculosis?

1. What do you think of the health system capacity to implement this home-based (IGRA) test for “hidden TB”?

(Probing questions)

- Enough infrastructure?
- Enough human resource?
- Transportation of samples
- Turnaround time of results?

1. What problems have you encountered in implementing this home-based (IGRA) test for “hidden Tuberculosis”?

(Probing questions)

- Inadequate supplies for the test?
- Shortage of staff to do the test?
- Fear for blood draw will keep away people?
- Sustainability challenges?
- Community or facility-based testing?

1. How do you think this blood-based IGRA test is more useful than what is currently being done to screen for “hidden tuberculosis”?

(Probing questions)

- Challenges of the current way of screening?
- How could the blood-based test overcome the above challenges?
- Identify individuals at risk more accurately & faster?

1. What final thing do you have to say about the home-based (IGRA) test for “hidden tuberculosis”?

(Probing questions)

- Should be adopted quickly or dropped?
- Need more information about the test?
- Would like to see this or that change in the way it was done in the study?
- More pressing priorities than introduction of this test?
