# Supplementary material for: Integrating interferon-gamma release assay testing into provision of tuberculosis preventive therapy is feasible in a tuberculosis high burden resource-limited setting: A mixed methods study
Source: PLOS Glob Public Health. 2022 Jul 6;2(7):e0000197. doi: 10.1371/journal.pgph.0000197 (PMC10022101; doi:10.1371/journal.pgph.0000197)
Supplement: Attachment — Submitted filename: Response to reviewers.docx [file pgph.0000197.s003.docx]

**Point by point response to reviewer comments**

**PGPH-D-21-00669**

**Study title:** IGRA based latent TB screening for provision of tuberculosis preventive therapy is feasible in a TB high burden resource limited setting: a mixed methods study

This is a significant topic for public health. LTBI screening and treatment is very important if the burden of TB is to be reduced and its relevance has been underestimated for decades in high-burden settings.
- Please include in the Abstract that IGRA were “home-based”. This is an important and distinctive contribution of this study that should be highlighted. The same comment for line 112 and 357

Response: Thank you for the comment. This has been addressed as guided.

- As mentioned by a reviewer, it is not clear if any of the participants from the qualitative research analysis were TB contacts or there were all health personnel. This need to be clarified and acknowledged in the study limitations.

Response: This has been clarified and acknowledged in the study limitations section line 505-508

- Line 89: IGRAs are more specific than TST but I am not sure it has been demonstrated that IGRA have more “sensitivity” than TST; please revise this statement

Response: The statement has been revised, line 95

- Methods: describe more clearly that TB contacts were sampled at home, and the conditions for blood samples transportation. Also please provide the version of the Quantiferon (Quantiferon TB-Gold or Quantiferon TB-Gold “In tube”?) and provider.

Response: we used QuantiFERON®-TB Gold Plus manufactured by QIAGEN QIAGEN-Gruupe, Germany. This has been added into the manuscript, line 149. A paragraph detailing on the sample transportation processes has also been included. Line 153-157.

Journal Requirements:

1. Please amend your detailed Financial Disclosure statement. This is published with the article, therefore should be completed in full sentences and contain the exact wording you wish to be published.

Response: This has been addressed as guided. Line 555-559

i). State the initials, alongside each funding source, of each author to receive each grant.

Response: This has been done as guided, line 557
ii). State what role the funders took in the study. If the funders had no role in your study, please state: “The funders had no role in study design, data collection and analysis, decision to publish, or preparation of the manuscript.”

Response: The funders had no role in the study. This has been stated in line 557
iii). If any authors received a salary from any of your funders, please state which authors and which funders.

Response: None of the authors received a salary from the funders. This has been stated under the financial disclosure statement. Line 558-559

2. In the online submission form, you indicated that "The datasets used and/or analyzed during the current study available from the corresponding author on request.". All PLOS journals now require all data underlying the findings described in their manuscript to be freely available to other researchers, either 1. In a public repository, 2. Within the manuscript itself, or 3. Uploaded as supplementary information.
This policy applies to all data except where public deposition would breach compliance with the protocol approved by your research ethics board. If your data cannot be made publicly available for ethical or legal reasons (e.g., public availability would compromise patient privacy), please explain your reasons by return email and your exemption request will be escalated to the editor for approval. Your exemption request will be handled independently and will not hold up the peer review process, but will need to be resolved should your manuscript be accepted for publication. One of the Editorial team will then be in touch if there are any issues.

Response: The dataset has been uploaded as supplementary information

3. Please provide separate figure files in .tif or .eps format only and remove any figures embedded in your manuscript file. Please ensure that all files are under our size limit of 20MB. Once you've converted your files to .tif or .eps, please also make sure that your figures meet our format requirements. For more information about how to convert your figure files please see our guidelines: <https://journals.plos.org/globalpublichealth/s/figures>

Response: Separate figure files are provided. They have been converted to .tif format as guided using PACE digital tool.

4. Please provide us with a direct link to the base layer of the map used in Fig1 and ensure this location is also included in the figure legend.

Please note that, because all PLOS articles are published under a CC BY license (creativecommons.org/licenses/by/4.0/), we cannot publish proprietary maps such as Google Maps, Mapquest or other copyrighted maps. If your map was obtained from a copyrighted source please amend the figure so that the base map used is from an openly available source.

Please note that only the following CC BY licences are compatible with PLOS licence: CC BY 4.0, CC BY 2.0  and CC BY 3.0, meanwhile such licences as CC BY-ND 3.0 and others are not compatible due to additional restrictions. If you are unsure whether you can use a map or not, please do reach out and we will be able to help you.

The following websites are good examples of where you can source open access or public domain maps:

* U.S. Geological Survey (USGS) - All maps are in the public domain. (<http://www.usgs.gov>)

* PlaniGlobe - All maps are published under a Creative Commons license so please cite “PlaniGlobe, <http://www.planiglobe.com>, CC BY 2.0” in the image credit after the caption. (<http://www.planiglobe.com/?lang=enl>)

* Natural Earth - All maps are public domain. (<http://www.naturalearthdata.com/about/terms-of-use/>)

**Response:** Thank you for the comment, this has been provided as guided. The base file (shapefile) for the maps was from openly available sources. It was downloaded from the Humanitarian Data Exchange portal <https://data.humdata.org/>”

The direct link to the shapefile is <https://data.humdata.org/dataset/uganda-administrative-boundaries-admin-1-admin-3>. This link has been added as a legend below the title for figure 1 within the manuscript

See below the description of the metadata for the shapefile including license

| **Source** | Uganda Bureau of Statistics with support from WHO |
| --- | --- |
| **Contributor** | [OCHA ROSEA](https://data.humdata.org/organization/37a0c6e3-3bcd-4457-aa81-6ddeb99dd203) |
| **Date of Dataset** | June 11, 2020-November 25, 2021 |
| **Updated** | 6 September 2021 |
| **Expected Update Frequency** | Every year |
| **Location** | [Uganda](https://data.humdata.org/group/uga) |
| **Visibility** | Public |
| **License** | [Creative Commons Attribution for Intergovernmental Organisations](https://data.humdata.org/faqs/licenses) |
| **Methodology** | Downloaded from the Uganda Bureau of Statistics [source](http://ubos.geo-solutions.it/layers/geonode:uganda_districts_2018). |
| **Caveats / Comments** | This COD-AB reflects the 135 district administrative system. |

5. Please review your reference list to ensure that it is complete and correct. If you have cited papers that have been retracted, please include the rationale for doing so in the manuscript text, or remove these references and replace them with relevant current references. Any changes to the reference list should be mentioned in the rebuttal letter that accompanies your revised manuscript. If you need to cite a retracted article, indicate the article’s retracted status in the References list and also include a citation and full reference for the retraction notice.

Response: The reference list is complete and correct. Some changes to the reference list have been made. That is, three additional references have been added;

- (Pamela B, Jack S. Qualitative Case Study Methodology: Study Design and Implementation for Novice Researchers. Qual Rep [Internet]. 2008 [cited 2020 Aug 18]; 13 (4): 544-59) to qualify the approach to the qualitative data analysis section. (reference #14)
- In response to Reviewer 1 comments #9 and #10 to expound on the discussion on acceptability of IGRA testing using the home-based approach and frame it in the literature. (references # 16 and #17)

Additional Editor Comments (if provided):

- Please change “GeneXpert” for Xpert MTB/RIF

Response: Resolved
- Please use abbreviations consistently (e.g. household contacts as HHC all over the text, same for LTBI)
- Please define “PR”

Response: PR stands for prevalence ratio. It has been written in full the first time it appears within the manuscript and abbreviated thereafter. Line 270

- Line 85: a comma is missing after ”supplies”

Response: Resolved, line 91

- Line 118: contacts were retested at 9 months, please revise as in Figure 2 indicates they were tested at 6 months. Was this also home-based testing?

Response: Thank you for the comment. Contacts were retested at 9 months. The error in Figure 2 has been corrected.

-Line 135: there is an extra period

Response: the extra period has been deleted, line 183

-Line 137: revise sentence writing

Response: Sentence writing has been revised

-Line 180: change the comma for a period

Response: This has been done as guided, thank you

- Line 192-193: revise sentence writing

Response: Thank you for pointing this out. It has been revised accordingly

- Line 203: there is an extra space after the parenthesis

Response: Resolved
- Results: the proportion of indeterminate results for both visits should be provided in the text (in Fig 1 states 1% at baseline and 11% in the second visit which is quite high, any explanation?)

Response: The proportion of indeterminate results for both visits have been provided in the text. Line 240

Explanation: this may have been due to sample transportation delays due to political riots during pre-election campaigns. This has been stated as a study limitation.

- Discussion line 392-303: revising the correct writing of "Mycobacterium tuberculosis"
Response: This has been corrected as guided, line 464

[Note: HTML markup is below. Please do not edit.]

Reviewers' comments:

Reviewer's Responses to Questions

**Comments to the Author**

1. Does this manuscript meet PLOS Global Public Health’s [publication criteria](https://eur01.safelinks.protection.outlook.com/?url=https%3A%2F%2Fjournals.plos.org%2Fglobalpublichealth%2Fs%2Fcriteria-for-publication&data=04%7C01%7Crmakabayi%40idi.co.ug%7Ccb3c84a80ae5469129d208d998d6dcdb%7C0d7c75a58002495ebb9f1f3108dcaa28%7C0%7C0%7C637708870916652202%7CUnknown%7CTWFpbGZsb3d8eyJWIjoiMC4wLjAwMDAiLCJQIjoiV2luMzIiLCJBTiI6Ik1haWwiLCJXVCI6Mn0%3D%7C1000&sdata=FeldqOFl1XOn7x4m%2B2ysXsNjV7OQVqwWFd3bZYn3o0U%3D&reserved=0)? Is the manuscript technically sound, and do the data support the conclusions? The manuscript must describe methodologically and ethically rigorous research with conclusions that are appropriately drawn based on the data presented.

Reviewer #1: Yes

Reviewer #2: Yes

2. Has the statistical analysis been performed appropriately and rigorously?

Reviewer #1: Yes

Reviewer #2: Yes

3. Have the authors made all data underlying the findings in their manuscript fully available (please refer to the Data Availability Statement at the start of the manuscript PDF file)?

The [PLOS Data policy](https://eur01.safelinks.protection.outlook.com/?url=https%3A%2F%2Fjournals.plos.org%2Fglobalpublichealth%2Fs%2Fdata-availability&data=04%7C01%7Crmakabayi%40idi.co.ug%7Ccb3c84a80ae5469129d208d998d6dcdb%7C0d7c75a58002495ebb9f1f3108dcaa28%7C0%7C0%7C637708870916662198%7CUnknown%7CTWFpbGZsb3d8eyJWIjoiMC4wLjAwMDAiLCJQIjoiV2luMzIiLCJBTiI6Ik1haWwiLCJXVCI6Mn0%3D%7C1000&sdata=z1tH93d7c2kwCyUySFIxB76cNjnjugRT3Ei59ZnkvZA%3D&reserved=0) requires authors to make all data underlying the findings described in their manuscript fully available without restriction, with rare exception. The data should be provided as part of the manuscript or its supporting information, or deposited to a public repository. For example, in addition to summary statistics, the data points behind means, medians and variance measures should be available. If there are restrictions on publicly sharing data—e.g. participant privacy or use of data from a third party—those must be specified.

Reviewer #1: Yes

Reviewer #2: No (Response: the dataset used for analysis has been added as part of supplementary information)

4. Is the manuscript presented in an intelligible fashion and written in standard English?

PLOS Global Public Health does not copyedit accepted manuscripts, so the language in submitted articles must be clear, correct, and unambiguous. Any typographical or grammatical errors should be corrected at revision, so please note any specific errors here.

Reviewer #1: Yes

Reviewer #2: Yes

5. Review Comments to the Author

Please use the space provided to explain your answers to the questions above. You may also include additional comments for the author, including concerns about dual publication, research ethics, or publication ethics. (Please upload your review as an attachment if it exceeds 20,000 characters)

Reviewer #1: This study sought to determine the feasibility of incorporating testing for LTBI using IGRA into the provision of TPT in Uganda. The authors conducted a cross-sectional study at four tertiary referral hospitals that were geographically distinct. The total population of household contacts identified was 355. Feasibility was defined as rate of uptake of the test. This is an area of research that could benefit from a stronger evidence base.

Overall, this was a well written paper describing both quantitative and qualitative results regarding the acceptance and feasibility of home-based LTBI testing. Greater discussion around some of the key points made from the FGD and KII would strengthen the discussion. A more robust discussion about the limitations of the sample size stratified by age groups would also strengthen the paper. Detailed comments below.

1. Methods, data collection section, line 107, the authors state that data collection was carried out as part of household contact investigation. Given contact investigation is implemented in a variety of ways, adding a few sentences about this contact investigation activity would give a better picture of the intervention.

Response: More details have been provided in the manuscript as guided

2. Methods, data collection section, line 109: the researchers are referred to as “we”. Who is we? A description of the study personnel and who is conducting the work at the household level would strengthen the methods.

Response: Details on the composition of the study team at the household level has been added in line 136-156

3. Results section, line 184: please describe selection and randomization process. This is an important piece of the study design that has not been detailed.

Response: Two sub-section on sample size determination (line 110-117) and participant selection process (118-131) have been added to the methods section of the manuscript to provide a detailed description of the process.

4. Table 1: The high number of participants (39.2%) in the 5-14 year old age group surprised me here as I was reading and I wanted to go back and review what the outcome measures for acceptability were. How would someone in the 5-14 year age group differ from an adult in reporting acceptability? Were there any age-specific measures for acceptability? How defining “acceptability” was done in the younger age group could be better described in the methods.

Response: We had no age specific measures for acceptability. It was the same across all study participants. This has been stated as a study limitation. Line 510-512.

5. Similar to the above comment, Table 2 shows that 14 of the 15 who reported poor acceptability were in the youngest age group. This needs to be addressed more clearly.

Response: Pain from the needle prick was cited as the main reason for poor acceptability. A statement has been added to clarify. Line 253-254 and line 441.

6. Table 2 shows that all of the 15 who reported poor IGRA acceptability were from the Hoima RRH health facility. Was this looked at more closely? Were there provider related factors influencing this finding?

Response: As noted by the reviewer, this could have resulted from provider related factors; however effort was made to train the study team comprised of qualified staff for 4 days on the study protocol and procedures prior to implementation including phlebotomy. Line 136-138

7. Prevalence of LTBI. Only 63.2% received repeat IGRA testing at 9 months. Are there any hypotheses for this loss to follow up? Please address.

Response: This was caused by Covid-19 related disruptions with some of the HHCs moving away from urban residences to rural areas for socio-economic reasons. This has been included in the discussion section of the manuscript. Line 490-492

8. Table 3: Under “contact proximity” there is a variable “different house”. Given this is defined as household contact investigation, please define this category more clearly.

Response: Thank you for the comment. This category has been defined as part of the legend below table 3 to further clarify. “Different house” depicts a typical scenario in rural areas characterized by homesteads that have adjacent outbuildings. The WHO LBTI guidelines define HHCs “a person who shared the same enclosed living space as the index case for one or more nights or for frequent or extended daytime periods during the three months before the start of current treatment”. Typically, despite the fact that HHCs in this setting may not sleep in the same house as the Index TB patient in this setting, they spend daytime periods together. This was described under section on study definitions.

9. Line 33: “The acceptability of the test was due, in part, to the fact that a homebased screening approach was employed which such that no transport costs were incurred in the process of receiving care.” This is a very important point to raise in the results, along with the accompanying quote. This point is not expanded on in the discussion, but I would think is a key piece to the “feasibility” question. Please expand on this and frame in the literature in the discussion.

Response: This has been addressed in the discussion section as guided above, line 442-448

10. Discussion: Line 403: “… IGRA based latent TB screening was well received by the community largely because it was free, and it was delivered at home.” I think this point links directly to comment #9 and should be expanded on, including addressing the question about sustainability of this intervention.

Response: This has been expanded on as per guidance, line 442-448. As noted in the recommendations, assessing the cost-effectiveness of the intervention should address the question about sustainability through a budget impact analysis as guided by reviewer #2.
Minor edits required:
Line 68: “falling” should read “fell” noted, correction has been done ad “in 2019” should be deleted as it is redundant. This has been deleted
Line 73: World Health Organization should be capitalized. This has been resolved
Line 84: “its but” should be deleted. This has been deleted, thank you
Line 244: Delete “constituted” Deleted
Line 390: replace “&” with “and” This has been replaced as indicated

Reviewer #2: Thank you for asking me to peer-review this manuscript. This is an important subject. LTBI screening and treatment is very important if the burden of TB is to be reduced but its importance is underestimated in the literature in high-burden settings.
This is a multicentre mixed methods study evaluating the uptake and acceptability of interferon-gamma release assay testing in a resource limited setting with a high incidence of tuberculosis. The study setting included four tertiary centres. The researchers recorded the uptake of IGRA-based LTBI screening among household contacts, aged over 5 years, of bacteriologically confirmed non-rifampicin resistant, non-multidrug resistant TB cases. IGRA screening was offered during a household visit. Active TB was excluded via a symptom screen. Data relating to duration and nature of contact of contacts with the case were collected also.

The qualitative component of this research comprised of focus groups performed at the tertiary centre and interviews with individuals from the regional centres. It would seem the qualitative component only included healthcare providers.

Response: Thank you for the comment, only health care providers were interviewed. Study participants were not interviewed in the qualitative component of this study. This has been stated as a study limitation. Line 506-508

I think the background requires more information for the reader to understand how the intervention (home-based IGRA screening) is different from typical practice. How are case contacts normally identified? Are contacts screened for LTBI as standard and if so how? Using TST? At the clinic or at home? By whom?

Response: More information has been provided in the background section of the manuscript, line 84-89.

In the introduction the authors state that the symptom screening tool is suboptimal for active TB exclusion. They go on to compare this with the TST. I found this confusing because in my mind it is drawing a comparison between a means of excluding active TB and a means of screening for LTBI.

Response: This has been clarified within the manuscript, line 84-89.

The authors go on to make valid comparisons between TST and IGRA use for LTBI screening. I think what is lacking from this comparison for the reader is how does uptake and acceptability of LTBI screening differ according to the test used in Uganda (or other high-incidence settings) among case contacts?

Response: More background information has been provided to clarify the Uganda context for LTBI screening and treatment, line 84-89.

Additionally, it should be noted that IGRA requires a laboratory and technician, trained phlebotomist and sample transport which may come at considerable cost compared with TST which may be more important in a resource limited setting. Again, I think some context as to what the current screening practice in Uganda is would help the reader determine what is new or different about this intervention in this setting.

Response: The current LBTI screening practice in Uganda has been added as mentioned above.

I’m not sure feasibility is the correct term either. Feasibility, in my mind incorporates more than acceptability but also cost, practicalities such as transport (I believe IGRA samples must reach the laboratory and tested within 24-36 hours for the test to be valid) etc. Perhaps, acceptability is a better descriptor as to what this study aimed to measure.
Response: recommendation line 520 has been changed to acceptability

The researchers say they randomly identified 86 index cases. I think it would be important to describe how these 86 cases were identified in the methods section where random selection is not mentioned. How many index TB patients were selected at random from which the 86 who agreed to participate arose? If the proportion of patients selected at random for enrolment who agreed to participate was low, then there is a risk of selection bias. Patients agreeing to participate probably are aware whether or not their household contacts would agree to LTBI screening, potentially biasing the uptake of IGRA testing.

Response: Details regarding the index TB patient selection process has been added to the manuscript. This has been explained as illustrated under reviewer #1 response to comment 3 above.

It would be good also to include a table of the TB patient characteristics to help the reader put into the context the population this study was performed in.

Response: A table has been added (*Table 4*). Details of the index TB patients had initially not been captured with focus mainly on HHCs. Effort was made to retrieve these records from Unit TB registers, however, due to administrative and site challenges, 53/86 records have been retrieved and reported in this submission.

Please include a sentence as to why patients with drug resistant TB were excluded. This may be obvious to the researchers but wasn’t clear to me.

Response: Drug resistant TB patients were excluded because they are not eligible for TPT according to the Uganda LTBI guidelines. The WHO (consolidated guidelines on tuberculosis 2020), however, recommends TPT use in HHCs of drug resistant TB patients in selected cases after evaluation of individual risk and clinical justification. This has been included in line 122-123

A second IGRA was performed at 9 months, presumably to capture those who seroconvert at a later date but is there a scientific basis for the 9-month cut-off chosen which should be cited here?
Response: That is correct, a second IGRA was repeated to capture those who seroconvert later. The repeat IGRA test was initially planned to be done at 6 months to rule out LTBI. As a result of Covid-19 travel restrictions, it was done at 9 months. This has been added in the discussion line 490-492.

Positivity rate for LTBI has been reported to be associated with increasing in age (time) (Ref: Kizza FN et al doi: 10.1186/s12879-015-0904-1) and increasing exposure duration (Ref: Reichler MR et al doi: 10.1093/cid/ciz1044. PMID: 32044987). In principle, we believe this would still yield valuable information and early treatment for those who turn out LTBI positive.

The qualitative study methods appear appropriate. Perhaps list the questions in the main text or an appendix.

Response: The questionnaire has been added as appendix 1

Data collection and analysis was assisted by members of the research team. Is there a risk that researchers who were involved in the conduct of the quantitative research could have been biased towards inferring positive experiences of the intervention from the transcripts?

Response: researchers trained research assistants and played a supervisory role. This has been clarified within the text, line 539-544.

Table 1: I suggest changing the layout. Under employment, 60.8% have none. Is this because they are in education? If so, the employment heading should consider only those not in education.
**Response:**

At data collection employment group included all those not in employment, both those in and out of school. But by further data exploration, we found that majority of those under category “None” for employment type, they were of school going age of 5 to 14 years (138/214 [64%]) as shown in summary below. We have added a statement in results section as “…. 61% had no employment of whom 64% (138/214) were children of school going age (5 to 14 years)” [line 245-246].

```
. tab employment agegrp, row m

+----------------+
| Key            |
|----------------|
|   frequency    |
| row percentage |
+----------------+

                  |    RECODE of ageofcontact (Ageofcontact)
       employment |      5_14      15_24      25_44        45+ |     Total
------------------+--------------------------------------------+----------
             None |       138         51         16          9 |       214
                  |     64.49      23.83       7.48       4.21 |    100.00
------------------+--------------------------------------------+----------
Formal employment |         0          3          9          3 |        15
                  |      0.00      20.00      60.00      20.00 |    100.00
------------------+--------------------------------------------+----------
         Business |         0         14         27          9 |        50
                  |      0.00      28.00      54.00      18.00 |    100.00
------------------+--------------------------------------------+----------
  Casual labourer |         0          7         12          2 |        21
                  |      0.00      33.33      57.14       9.52 |    100.00
------------------+--------------------------------------------+----------
      Agriculture |         0          9         27         16 |        52
                  |      0.00      17.31      51.92      30.77 |    100.00
------------------+--------------------------------------------+----------
            Total |       138         84         91         39 |       352
                  |     39.20      23.86      25.85      11.08 |    100.00
```

Test uptake was exceptionally high, so too was reports of test experience as good/excellent. The authors say older age was associated with a bad phlebotomy experience. Should this not be younger age?

Response: This is correct, it should be the younger age. This statement has been clarified.

Additionally, all those who had a negative phlebotomy experience were in one setting. This to me suggests it was related more to the setting. Was the phlebotomist here perhaps not as good at performing phlebotomy in children compared with the other hospitals? Could any inisghts be drawn on this from the qualitative analysis?
Response: This would have been captured from the perspective of the patients through qualitative interviews. This was noted as a study limited as noted in an earlier comment

Regarding factors associated with a positive IGRA, multiple analyses are performed. These analyses appear to me to be exploratory and so it might be good to indicate in the methods section that all these analyses were not corrected for the multiplicity given the exploratory nature of the study.

**Response**:

We have added the suggested statement in methods section, that “analyses were not corrected for multiplicity given the exploratory nature of the study” [line 214-215].

Additionally, depending on journal style, it may not be necessary or informative to include all P-values, particularly given the large number of analyses. Aside from this the factor identified as being associated with LTBI are logical and therefore believable and less likely to false positives. However, given the aim of the study was to determine the uptake and acceptability of the test is inclusion of these analyses even necessary? If so, I would suggest adding a sentence in the introduction to state this is a secondary aim.
 **Response:**

We agree that there is multiplicity and did not adjust the P-values for multiple hypotheses testing. We have however added a statement under methods that *“.. analyses were not corrected for multiplicity given the exploratory nature of the study*” [line 214-215].

The qualitative analysis was one third laboratory staff, would seem over represented to me and a limitation.

Response: It is a lab-based intervention that calls for more lab personnel, line 180-182 added in the text

Additionally, the qualitative analysis included none of the patients. The intervention seemed acceptable to the staff involved and they perceived it to be acceptable in general to the patients. It should be noted in the discussion the exclusion of patients was a weakness of the study design because some of the findings could not be explored further without their input e.g., the mentions of stigma by the healthcare workers and the high uptake and acceptability. Ultimately this study does not explain these further from the patients’ perspective. The healthcare workers seemed to think it was the avoidance of travel was the reason uptake was high but without asking the patients this cannot be said for sure. It may have been other factors related to the clinic or the homeplace or the behaviour of the staff (the staff had a positive experience, perhaps enthusiastic staff were more persuasive than they would have been in a clinic setting).
Response: We acknowledge that perspectives of patients were not included in this study. This has been stated as a study limitation in the discussion.

The discussion is acceptable.

Response: Thank you

Recommendations- I think it would be more correct to say “targeted IGRA screening for household contacts is acceptable” rather than “feasible” and the authors have gone on to say further evaluations are needed to determine its cost effectiveness (and they should add a budget impact analysis).
Response: this has been corrected as guided.

Some spelling and grammatical errors throughout.
Response: revisions have been made throughout the document

To summarize, a well conducted mixed methods study on LTBI screening uptake and acceptability, an important subject for TB elimination globally. More information needed on what the current practice is regarding LTBI screening of contacts in Uganda (if any LTBI screening). More information on how the 86 cases were selected and their characteristics. Acceptability only evaluated from healthcare providers perspective which should be discussed as a major limitation.

Response: Thank you for the review summary, these have been addressed

6. PLOS authors have the option to publish the peer review history of their article ([what does this mean?](https://eur01.safelinks.protection.outlook.com/?url=https%3A%2F%2Fjournals.plos.org%2Fglobalpublichealth%2Fs%2Feditorial-and-peer-review-process%23loc-peer-review-history&data=04%7C01%7Crmakabayi%40idi.co.ug%7Ccb3c84a80ae5469129d208d998d6dcdb%7C0d7c75a58002495ebb9f1f3108dcaa28%7C0%7C0%7C637708870916672191%7CUnknown%7CTWFpbGZsb3d8eyJWIjoiMC4wLjAwMDAiLCJQIjoiV2luMzIiLCJBTiI6Ik1haWwiLCJXVCI6Mn0%3D%7C1000&sdata=azlTyBSZX9%2F6Rt5vRkEsmnq2IjET7rE%2B6uZeKkdiqi4%3D&reserved=0)). If published, this will include your full peer review and any attached files.


**Do you want your identity to be public for this peer review?** If you choose “no”, your identity will remain anonymous but your review may still be made public.


For information about this choice, including consent withdrawal, please see our [Privacy Policy](https://eur01.safelinks.protection.outlook.com/?url=https%3A%2F%2Fwww.plos.org%2Fprivacy-policy&data=04%7C01%7Crmakabayi%40idi.co.ug%7Ccb3c84a80ae5469129d208d998d6dcdb%7C0d7c75a58002495ebb9f1f3108dcaa28%7C0%7C0%7C637708870916672191%7CUnknown%7CTWFpbGZsb3d8eyJWIjoiMC4wLjAwMDAiLCJQIjoiV2luMzIiLCJBTiI6Ik1haWwiLCJXVCI6Mn0%3D%7C1000&sdata=orPPK5674OMXOdJ6JWCDIqv%2BIh0ouQyThX9kSfIlaP4%3D&reserved=0).

Reviewer #1: No

Reviewer #2: **Yes:** James O'Connell

[NOTE: If reviewer comments were submitted as an attachment file, they will be attached to this email and accessible via the submission site. Please log into your account, locate the manuscript record, and check for the action link "View Attachments". If this link does not appear, there are no attachment files.]

While revising your submission, please upload your figure files to the Preflight Analysis and Conversion Engine (PACE) digital diagnostic tool, <https://pacev2.apexcovantage.com/>. PACE helps ensure that figures meet PLOS requirements. To use PACE, you must first register as a user. Registration is free. Then, login and navigate to the UPLOAD tab, where you will find detailed instructions on how to use the tool. If you encounter any issues or have any questions when using PACE, please email PLOS at [figures@plos.org](mailto:figures@plos.org). Please note that Supporting Information files do not need this step.

Response: This website was accessed, and files converted as guided to TIF files.

*In compliance with data protection regulations, you may request that we remove your personal registration details at any time.* [*(Remove my information/details)*](https://eur01.safelinks.protection.outlook.com/?url=https%3A%2F%2Fwww.editorialmanager.com%2Fpgph%2Flogin.asp%3Fa%3Dr&data=04%7C01%7Crmakabayi%40idi.co.ug%7Ccb3c84a80ae5469129d208d998d6dcdb%7C0d7c75a58002495ebb9f1f3108dcaa28%7C0%7C0%7C637708870916682185%7CUnknown%7CTWFpbGZsb3d8eyJWIjoiMC4wLjAwMDAiLCJQIjoiV2luMzIiLCJBTiI6Ik1haWwiLCJXVCI6Mn0%3D%7C1000&sdata=KjsZKWV9DCmpdheAQ83Xb9vdZUnHReFR4ZEhnQaIuXA%3D&reserved=0)*. Please contact the publication office if you have any questions.*
