# Supplementary material for: Integrating interferon-gamma release assay testing into provision of tuberculosis preventive therapy is feasible in a tuberculosis high burden resource-limited setting: A mixed methods study
Source: PLOS Glob Public Health. 2022 Jul 6;2(7):e0000197. doi: 10.1371/journal.pgph.0000197 (PMC10022101; doi:10.1371/journal.pgph.0000197)
Supplement: Attachment — Submitted filename: Response to Reviewers.docx [file pgph.0000197.s004.docx]

**Response to Reviewers**

PGPH-D-21-00669R1
IGRA based latent TB screening for provision of tuberculosis preventive therapy is feasible in a TB high burden resource limited setting: a mixed methods study
PLOS Global Public Health

Journal Requirements:

1. We have noticed that you have uploaded supporting information, but you have not included a list of legends. Please add a full list of legends for all supporting information files (including figures, table and data files) after the references list.

Response: the IGRA study dataset was submitted as supporting information. The legend for the supporting information has been added after the references list of the manuscript, line 653.

2. Please review your reference list to ensure that it is complete and correct. If you have cited papers that have been retracted, please include the rationale for doing so in the manuscript text, or remove these references and replace them with relevant current references. Any changes to the reference list should be mentioned in the rebuttal letter that accompanies your revised manuscript. If you need to cite a retracted article, indicate the article’s retracted status in the References list and also include a citation and full reference for the retraction notice.

Response: the reference list is complete and accurate to best of our knowledge. No papers cited have been retracted. No change(s) has been made to the reference list.

Additional Editor Comments (if provided):

Thanks authors for their great effort to improve the manuscript. The paper improved but there remain some issues that should be resolved.

ABSTRACT
• The current Abstract is too long, it must not exceed 300 words: <https://journals.plos.org/globalpublichealth/s/submission-guidelines>

Response: we thank the reviewer for the comment. The submission system allowed for 500 words hence the error. However, the abstract word count has been reduced accordingly in line with the submission guidelines.

• line 32: “that would require TPT was unknown”: change to ”that would require TPT is unknown”

Response: this has been resolved.

• line 38: “without signs or symptoms of TB”: change to ”without signs of symptoms of active TB”

Response: this has been resolved

• line 52 (and also in line 269): “older age >45”: “>45 years old”…

Response: this has been corrected, thank you.

Response:
• line 59: “for sample storage”: change to ”for blood sample storage”…

Response: resolved

• line 61: ”IGRA based”: change to ”Home-based IGRA screening”…

Response: resolved as guided

• line 86 (and again at line 119): “PBCs” Is undefined (please avoid too many abbreviations)

Response: Apologies, PBCs has been defined

• Line 51-52: IGRA negative results testing positive at 9 months are 4.3%, but in Results section of the manuscript, the same proportion is reported as 3.4% (line 265). Which is the correct?

Response: Thank you for identifying this discrepancy. The correct proportion is 3.4% and has been documented accordingly.

INTRODUCTION
• lines 81-89: Please reformulate, as the new paragraph is a little bit confusing regarding what is recommended to screen active TB versus latent TB. Furthermore, the sentence “Although TST has better sensitivity and specificity than the WHO symptom screen,” is not correct as the symptom screening algorithm is purposed to find active TB, and TST is for finding LTBI. Additionally, what is currently done as standard of care for HHC in Uganda is still unclear in the Introduction.

Response: This section has been revised and standard care for HHC in Uganda clarified.

• IGRA: there are more than one IGRA available on the market, so I suggest changing to plural (IGRAs) when referring to any IGRA.

Response: This has been changed in relevant sections throughout the document.

METHODS
• A sample size calculation for survey is now provided in the corrected version of the manuscript. However, the authors subtract 10% accounting for those HHC that may be found later having active TB. I think I would do the opposite (adding 10% to account for those that will have to be excluded). Unless you assume that the n=385 already includes only those fulfilling inclusion and exclusion criteria, in which case you may just eliminate the sentence line 113-114. Also, revise spelling and clarity of the sentences (e.g “formular” in line 110), please refer to standard way of reporting sample sizes calculations.

Response: We thank the reviewers for the comment. We acknowledge the error made in the sample size statement. This section has been re-written, and clarifications made. In line with this, a study limitation has been added within the discussion section of the manuscript.

• Line 119: “Of these 299 patients...”: change to: “Of these, 299 patients..”

Response: resolved

• Line 127: “One index TB patients declined” : removed the “s” from patients

Response: resolved

• Line 133-134: It is important to describe how the 86 index cases were randomly selected (what was the proportion for all TB cases found in the same period Feb to Dec 2020)

Response: This has been clarified to reflect the fact that Index TB patients who were pulmonary bacteriologically confirmed during the period of three month prior to study commencement were included in the study. The period of February to December 2020 involved HHCs.

RESULTS
• As previously raised by one of the reviewers, children under 18 should not be included in the “unemployed category”. Employment status or type should rather include only those not on education (Table 1)

Response: Thank you for the comment. This has been addressed as shown in table 1 line 236 to 237

• Line 287-292 and Table 4. It should include which was the proportion of all index cases included in this study for which information was available (53 out of 86)

Response: the proportion has been included as guided by the reviewer line 280 and line 285-286.

DISCUSSION
• Line 448: “the prevalence determined in this study”: change to ”the prevalence of latent TB infection (or LTBI) determined in this study”

Response: resolved
• Line 450-451: “studies were carried out urban or pre-urban setting”: change to ”studies were carried out in urban or pre-urban setting”

Response: resolved

• “Further,...”: change to: “Furthermore,...”

Response: resolved
• Line 507-508: “high rates of indeterminate results were reported”: change to “high rates of indeterminate IGRA results were reported”

Response: resolved

• Line 509-511: the sentence comment on age-specific measures to increase acceptability could be introduced by : Finally,… (as it is not related to the previous limitation in the text), or maybe moved up in the same paragraph (e.g. at the end of line 502).

Response: thank you for the comment, this has been resolved in accordance with the guidance

CONCLUSION:
• Line 514: “IGRA based screening had a high uptake and acceptability and therefore feasible in …”: change to ”Home-based IGRA screening for latent tuberculosis infection had a high uptake and acceptability and therefore is feasible in …”

Response: resolved as per guidance provided

ALL OVER THE TEXT
• When “interferon gamma release assay”: change to “interferon-gamma release assay”

Response: this has been adjusted accordingly throughout the manuscript

• “IGRA QuantiFERON-TB Gold Plus test” is a little long. I suggest to abbreviate as QFT or QFT-G. And it is not necessary to put both together, just IGRA or just QFT depending on context (but not “IGRA QuantiFERON-TB Gold Plus”

Response: this has been resolved as guided using abbreviation QFT-Plus or IGRA depending on context

• Revise once again the consistency of all abbreviations (LTBI, TB, PTB, etc) all over the text

Response: revised as guided, thank you.

Response:
• “IGRA based”: change to “IGRA-based”

Response: resolved

Response:
• “homebased” or “home base”: change to ”home-based”

Response: revised accordingly


[Note: HTML markup is below. Please do not edit.]

Reviewers' comments:


[NOTE: If reviewer comments were submitted as an attachment file, they will be attached to this email and accessible via the submission site. Please log into your account, locate the manuscript record, and check for the action link "View Attachments". If this link does not appear, there are no attachment files.]

While revising your submission, please upload your figure files to the Preflight Analysis and Conversion Engine (PACE) digital diagnostic tool, <https://pacev2.apexcovantage.com/>. PACE helps ensure that figures meet PLOS requirements. To use PACE, you must first register as a user. Registration is free. Then, login and navigate to the UPLOAD tab, where you will find detailed instructions on how to use the tool. If you encounter any issues or have any questions when using PACE, please email PLOS at [figures@plos.org](mailto:figures@plos.org). Please note that Supporting Information files do not need this step.

*In compliance with data protection regulations, you may request that we remove your personal registration details at any time.* [*(Remove my information/details)*](https://eur01.safelinks.protection.outlook.com/?url=https%3A%2F%2Fwww.editorialmanager.com%2Fpgph%2Flogin.asp%3Fa%3Dr&data=04%7C01%7Crmakabayi%40idi.co.ug%7C1957cf94a24749503d2908d9e5b82799%7C0d7c75a58002495ebb9f1f3108dcaa28%7C0%7C0%7C637793401376960614%7CUnknown%7CTWFpbGZsb3d8eyJWIjoiMC4wLjAwMDAiLCJQIjoiV2luMzIiLCJBTiI6Ik1haWwiLCJXVCI6Mn0%3D%7C2000&sdata=xPNXb7h%2BMga7HbwXI7vXlc3wBo%2BLIi70qRgk0VEZQgs%3D&reserved=0)*. Please contact the publication office if you have any questions.* *** This messages originates from outside IDI. Click on links and attachments if sure of the source ***
