# Supplementary material for: Integrating interferon-gamma release assay testing into provision of tuberculosis preventive therapy is feasible in a tuberculosis high burden resource-limited setting: A mixed methods study
Source: PLOS Glob Public Health. 2022 Jul 6;2(7):e0000197. doi: 10.1371/journal.pgph.0000197 (PMC10022101; doi:10.1371/journal.pgph.0000197)
Supplement: Attachment — Submitted filename: Response to Reviewers.docx [file pgph.0000197.s005.docx]

Response to Reviewers

PGPH-D-21-00669R2
Integrating Interferon Gamma Release Assay testing into provision of tuberculosis preventive therapy is feasible in a TB high burden resource limited setting: a mixed methods study
PLOS Global Public Health

Dear Dr. Makabayi-Mugabe,

Thank you for submitting your manuscript to PLOS Global Public Health. After careful consideration, we feel that it has merit but does not fully meet PLOS Global Public Health’s publication criteria as it currently stands. Therefore, we invite you to submit a revised version of the manuscript that addresses the points raised during the review process.

Please submit your revised manuscript by Jul 08 2022 11:59PM. If you will need more time than this to complete your revisions, please reply to this message or contact the journal office at [globalpubhealth@plos.org](mailto:globalpubhealth@plos.org). When you're ready to submit your revision, log on to <https://www.editorialmanager.com/pgph/> and select the 'Submissions Needing Revision' folder to locate your manuscript file.

Please include the following items when submitting your revised manuscript:

- A rebuttal letter that responds to each point raised by the editor and reviewer(s). You should upload this letter as a separate file labeled 'Response to Reviewers'.
- A marked-up copy of your manuscript that highlights changes made to the original version. You should upload this as a separate file labeled 'Revised Manuscript with Track Changes'.
- An unmarked version of your revised paper without tracked changes. You should upload this as a separate file labeled 'Manuscript'.

Guidelines for resubmitting your figure files are available below the reviewer comments at the end of this letter.

We look forward to receiving your revised manuscript.

Kind regards,

María Elvira Balcells, M.D., MSc
Academic Editor
PLOS Global Public Health

Journal Requirements:

1. Please update your Competing Interests statement. If you have no competing interests to declare, please state: “The authors have declared that no competing interests exist.”

Response: Resolved as guided.

2. Please provide an Author Summary. This should appear in your manuscript between the Abstract (if applicable) and the Introduction, and should be 150–200 words long. The aim should be to make your findings accessible to a wide audience that includes both scientists and non-scientists. Sample summaries can be found on our website under Submission Guidelines: <https://journals.plos.org/globalpublichealth/s/submission-guidelines#loc-parts-of-a-submission>
Alternative link: <http://journals.plos.org/ploscompbiol/s/submission-guidelines#loc-author-summary>

Response: An author summary has been added as per guidance.

3. Please review your reference list to ensure that it is complete and correct. If you have cited papers that have been retracted, please include the rationale for doing so in the manuscript text, or remove these references and replace them with relevant current references. Any changes to the reference list should be mentioned in the rebuttal letter that accompanies your revised manuscript. If you need to cite a retracted article, indicate the article’s retracted status in the References list and also include a citation and full reference for the retraction notice.

Response: To the best of our knowledge, the reference list is complete and correct.

Additional Editor Comments (if provided):

1. Title:
Replace the abbreviation “TB” with “tuberculosis”; “interferon gamma” with “interferon-gamma” (revise this in all the manuscript); “resource-limited” with “resource-limited”

Response: This has been resolved as guided

2. Abstract:
• Line 34: I suggest changing “pulmonary bacteriologically confirmed tuberculosis” to “bacteriologically confirmed pulmonary tuberculosis” (the same for Table 4)

Response: Resolved as guided
• Please restore the measures of association (e.g. PR and 95%CI) for factors significantly associated with LTBI in the Abstract

Response: Thank you for the comment, the measures of association were erroneously removed in an effort to reduce the word count. We do acknowledge that they provide better understanding of our research findings in quantitative terms. They have been restored as guided.
• Also, why other factors significantly associated with LTBI such as the type of work were removed from the abstract?

Response: These factors have been restored
• The authors removed the word “home-based” from Abstract conclusion, I suggest re-including it, as this is an important novelty of this study

Response: We thank the reviewers for the comment. The word “home-based” has been re-included in the abstract conclusion.

3. Introduction:
• Review this sentence line 71-72: “Although immunological tests e.g.,the Tuberculin Skin Test (TST) have better sensitivity and specificity than the WHO symptom screen,…” (“as” is lacking before “the TST”?)
Response: “as” has been added as guided
4. Methods:
• Line 86-87: Please provide the name of participating hospitals centers

Response: the names of participating hospital centers have been added
• Line 107 states “Drug resistant TB patients were not eligible for TPT…” Please correct to “HHCs of drug-resistant TB patients were not eligible for TPT…”

Response: this has been corrected
• Sample size: 106 index cases for 424 HHCs were required. The authors state that only 86 index cases and 352 HHCs were enrolled “due to limited availability of test kits”. Therefore, revise line 115 as the sentence “Consequently, 86 index TB case’s homes were visited…” does not make it very clear at which point or which of the four participating hospitals did not complete the sample size. Was the sampling proportionate to size maintained?
Response: this has been re-written for clarity. We maintained the sampling proportionate to size.
5. Results
• Spelling revise and correct to: Line 223 “follow-up”; line 229 “the” majority; line 241 “p value =0.02”); line 243 “unadjusted”

Response: Resolved as guided, thank you.
• Table 4: format the table results as previous tables; e.g. the percentages are included in the same column as the absolute numbers, e.g. 14(26.4%). The dashs for age intervals should be at the middle, e.g 15-17 and not 15_17, >45 and not 45+, etc. Line 281(Table footnote) revise the sentence for clarity.

Response: Resolved as guided.
• Line 288-289: “we carried out two focus group discussions (FGDs) each with five participants and 14 key informant interviews (KII) giving a total of 24 participants” (5+14=19, not 24…please revise).

Response: Kindly re-visit. A comma has been added for clarity but no change has been made to total number of health worker participants. Total number stated is correct. It is ((5x2) +14) =24.

Also, importantly, please change “participant” for “health care worker participants in this study “ (or something alike) to not confuse readers with HHCs participants.

Response: We thank the reviewers for this comment. This has been re-stated to further clarify the study participant category under study in this section.

6. Discussion
• Line 437 “Tuberculin Skin Test” was already defined as “TST” in previous paragraphs.

Response: Resolved
• Line 447 introducing M.tb abbreviation seems unnecessary here

Response: This has been removed
• Line 448 change “latent TB infection outside the household…” for “latent TB infection acquisition outside the household…”

Response: Thank you for the addition, this has been resolved.
7. Funding: line 545 states “None of the authors received a salary from the authors.” Should it be “…. salary from the funders”?

Response: Resolved. We thank the reviewers for the detailed review of the manuscript.
[Note: HTML markup is below. Please do not edit.]

Reviewers' comments:


[NOTE: If reviewer comments were submitted as an attachment file, they will be attached to this email and accessible via the submission site. Please log into your account, locate the manuscript record, and check for the action link "View Attachments". If this link does not appear, there are no attachment files.]

While revising your submission, please upload your figure files to the Preflight Analysis and Conversion Engine (PACE) digital diagnostic tool, <https://pacev2.apexcovantage.com/>. PACE helps ensure that figures meet PLOS requirements. To use PACE, you must first register as a user. Registration is free. Then, login and navigate to the UPLOAD tab, where you will find detailed instructions on how to use the tool. If you encounter any issues or have any questions when using PACE, please email PLOS at [figures@plos.org](mailto:figures@plos.org). Please note that Supporting Information files do not need this step.

*In compliance with data protection regulations, you may request that we remove your personal registration details at any time.* [*(Remove my information/details)*](https://eur01.safelinks.protection.outlook.com/?url=https%3A%2F%2Fwww.editorialmanager.com%2Fpgph%2Flogin.asp%3Fa%3Dr&data=05%7C01%7Crmakabayi%40idi.co.ug%7C69a9de2436d94584f69408da3d6c7bf9%7C0d7c75a58002495ebb9f1f3108dcaa28%7C0%7C0%7C637889833389686116%7CUnknown%7CTWFpbGZsb3d8eyJWIjoiMC4wLjAwMDAiLCJQIjoiV2luMzIiLCJBTiI6Ik1haWwiLCJXVCI6Mn0%3D%7C3000%7C%7C%7C&sdata=VPCVRM48X7Sv%2BgZzwdcVktC3ByA2KGnIM0K7QlXQEvc%3D&reserved=0)*. Please contact the publication office if you have any questions.* ***
